# Time trends in gender-specific incidence rates of road traffic injuries in Iran

**DOI:** 10.1371/journal.pone.0216462

**Published:** 2019-05-09

**Authors:** Milad Delavary Foroutaghe, Abolfazl Mohammadzadeh Moghaddam, Vahid Fakoor

**Affiliations:** 1 Department of Civil Engineering, Faculty of Engineering, Ferdowsi University of Mashhad, Mashhad, Razavi Khorasan, Iran; 2 Department of Statistics, Faculty of Mathematics, Ferdowsi University of Mashhad, Mashhad, Razavi Khorasan, Iran; University of Iowa, UNITED STATES

## Abstract

**Objectives:**

Every day, an average of 3,400 deaths and tens of millions of injuries occur as a result of traffic accidents. This study aims to model and validate road traffic injury (RTI) times series, specifically considering gender.

**Study design:**

Time trend studies of monthly road traffic injuries (RTI) in Iran from March 2005 to February 2016, as well as those of males and females from March 2009 to February 2016 were performed.

**Methods:**

The seasonal auto-regressive integrated moving average method (SARIMA) was employed to predict RTI time series. The final model was selected from various SARIMA models based on the Akaike information criterion (AIC) and the Bayesian information criterion (BIC). To examine whether the residuals were white noise, the Ljung-Box (LB) test and residuals plots were used for un-correlation, and the zero mean and stationarity, respectively. Additionally, smoothing methods were utilized to validate the SARIMA models for fitting and out-of-range prediction of the time series models under study. The sample auto-correlation function (ACF) and the partial autocorrelation function (PACF) with 20 lags were employed to determine the order of models and to ascertain if the residuals of the model were uncorrelated.

**Results:**

Based on the obtained results, SARIMA (2,1,0)(0,1,1)_12_, SARIMA (0,1,1)(0,1,1)_12_, SARIMA (1,1,1)(0,0,1)_12_, and SARIMA (2,0,0)(1,0,0)_12_ were chosen for the time series including incidence rates of total road traffic injuries (IRTI), IRTI of males, females, and males-to-females, respectively. The AIC values were -87.57, 413.38, -732.91, and -85.32, respectively. The LB test for the residuals of the time series models of (0.539) IRTI, (0.3) IRTI of males, (0.23) females, and (0.237) males-to-females indicated that residuals were uncorrelated. Furthermore, prediction values for the next 24 months (2016 to 2018) showed no decline in the incidence rate of male and female traffic injuries. Results of the predictions using exponential smoothing methods indicated out-of-range prediction validity of the SARIMA models.

**Conclusion:**

This study exemplified the high efficiency of SARIMA models in predicting road traffic injuries (RTIs). Based on observations, the IRTI mean in Iran was 35.57 in 2016. The predicted values of the IRTI for 2016–2018 by the SARIMA model showed no decreasing trend. During the studied period, the observed values of IRTI for males were two to three times the female values. Thus, prediction of RTI can provide a useful tool for traffic safety policymaking by simulating interrupted time series when applying new traffic enforcement interventions and regulations in the future. Additionally, IRTI analysis of males and females showed that men had a non-increasing trend but higher incidence of traffic injuries, whereas the IRTI for women revealed an increasing trend from 2009 to 2012 with a lower incidence of injuries. This growth could be attributed to the impact of increased outdoor activities of women and the increased number of issued driving licenses in the period of 2009–2012.

## 1. Introduction

Road traffic accidents present serious challenges in public health all over the world. Road traffic mortality (RTM), road traffic injuries (RTIs), and disabilities are among the top concerns in this regard [1]. Traffic injuries refer to the number of people who were bodily injured in a traffic accident. The level of injury or property damage due to a crash is referred as “crash severity” in the highway safety manual. Crash severity is often divided into categories according to the KABCO scale, which provides five levels of injury severity, including fatal injury, incapacitating injury, non-incapacitating evident injury, possible injury, and no injury/property damage only (PDO). However, in this study the word “injuries” refers to the number of incapacitating injuries, non-incapacitating evident injuries, and possible injuries due to traffic accidents [2]. Annually, over 1.25 million individuals lose their lives in road traffic accidents around the world. RTIs are the main cause of death for people aged 15 to 29 years. As reported by the World Health Organization (WHO) in 2018, road traffic accidents cost 3% of the gross domestic product (GPD) for most countries. If no actions are taken against it, it could well become the seventh highest cause of death by 2030 worldwide [3].

Studies conducted in developing countries indicated that 85% of deaths and 90% of disabilities were related to road traffic accidents. As estimated by WHO, there will be a 27% global decline in road traffic accidents up to 2020; however, accidents are predicted to increase by 80% in developing countries [4, 5]. Therefore, the pattern for mortality and injuries caused by traffic accidents in developing countries will be different to that of the developed countries, which demands dedicated models and strategies for improving prediction accuracy [6]. Furthermore, active interventional policymaking should be implemented as the main factor for reducing RTMs and RTIs [7].

To provide statistics on RTI incidence rate, i.e., the number of injured people in every 100,000 monthly (mid-month population), was used. According to the statistics collected from the Iranian legal medicine website, IRTI for 2015 and 2016 were 32.57 and 25.57, respectively, and there has been no reduction or increase in the number of RTIs since 2005 [8]. In addition, 500 people a day were bodily injured in traffic accidents, based on statistics published by the Iranian traffic police. However, the reports of traffic police were not completely accessible in Iran [9].

Different methods, such as SARIMA, were employed to model time series in time domains, because a single ARIMA model cannot analyze the seasonal features of data [10–12]. Due to its predictability and rich information in time-dependent changes, the SARIMA model demonstrated superiority over other predictive methods, including exponential smoothing, moving average, artificial neural network, and fuzzy logic [10, 13]. In a study conducted to compare the predictive behavior of dynamic linear models and SARIMA in the field of epidemiology, both models were comparable based on an accuracy assessment [14]. A study comparing the accuracy of SARIMA models, Poisson regression of time series, and decomposition showed better performance from the SARIMA model [15, 16].

One of the disadvantages of the SARIMA model is the application of longitudinal data with a large size. The number of data points necessary for time series modeling is at least 80, which requires seven years to be collected given the availability of monthly data [11]. In another study, Nobre et al. determined that at least 52 data points were necessary to develop a time series model [14]. Therefore, one of the reasons for applying time series models such as SARIMA in epidemiological studies is the existence of a minimum number of points for longitudinal studies in identifying factors affecting the incidence and prevalence [17].

Bahadordi et al. [18] and Zhang et al. [6] utilized SARIMA(0,1,1)(0,1,1)_12_ and SARIMA(1,1,1)(0,1,1)_12_ models to predict the RTM in Iran and China, respectively. SARIMA models in epidemiology have also been used to predict the evolution of infectious diseases, including deaths from pneumonia [19], influenza [20], malaria, and hepatitis in the United States [14] and urban malaria in Calcutta [21]. One of the applications of SARIMA models is to investigate the impact of interventions in addition to forecasting the future according to the Box-Jenkins algorithm [22]. Traffic law enforcement is an example of an intervention, which can affect response variables such as number of casualties or injuries. The interventional analysis is useful when the exact effect of interventions is of interest or the aim of the analysis is to predict the time series by applying the effect of the intervention. For instance, laws related to U.S. child safety seats have been investigated over the past 35 years, saving up to 39 children per year [23]. Lee et al. [24] evaluated the impact of marijuana restrictions on marijuana-involved fatal crashes in the United States in 2018, noting no significant changes in the number of marijuana-related crashes after medical legalization.

Ajdacic-Gross et al. [25] used the SARIMA model with an interventional effect to evaluate the relationship between birth and death days on Swedish mortality data between 1969 and 2008. Grundy et al. [26] utilized an interrupted time series analysis method to investigate the effect of creating 20 mph traffic speed zones on RTIs and RTM in London between 1986 and 2006. Nistal-Nuño [27] evaluated the impact of a reduction in the legal blood alcohol concentration (BAC) while driving on mortality and morbidity from January 2003 to December 2014. The present study was designed to determine SARIMA models for IRTI, IRTI of male, female, and males-to-females, along with providing a validation for each of the models using exponential smoothing methods.

In addition to ACF and PACF plots, tests such as Keenan and Augmented Dickey-Fuller (ADF-GLS) were applied to examine linearity and stationarity of time series in SARIMA, respectively. Furthermore, the main condition for the acceptance of SARIMA models (i.e., residuals being white noise) was studied through an LB test for lack of correlation and the residuals plots for the condition of zero mean and variance stationarity. In addition to the main condition of white noise, the normal distribution of residuals was also utilized as an optional condition for examining these models.

Incidence and prevalence are two major concepts frequently used in the epidemiology field of epidemic diseases. Therefore, considering the global prevalence of RTIs and RTM, the concept of incidence rate was employed in this study. Because the IRTI in Iran showed no decline and was high compared to other countries, it was vital to model and predict IRTI in order to help efficiently allocate funds and develop or modify interventional policies in road traffic safety. In Iran, the prevalence of outdoor activities for men is higher than for women, based on culture. One of the outputs of the current study was investigating the gender-based road traffic injuries in Iran. This also determined that the male trend and quantity of IRTIs were higher than those of females.

## 2. Methodology

### 2.1. Data collection

The data required to conduct this study, including the total monthly number of RTIs in Iran from March 2005 to February 2016, as well as male and female genders from March 2009 to February 2016, were collected from the Legal Medicine Organization of Iran website. RTI data were published by this organization for each province [8]. Therefore, all the data concerning injuries caused by traffic accidents in Iranian provinces were gathered in order to obtain the final time series. To analyze and predict the future status of IRTI of males-to-females in Iran, this ratio was calculated monthly and utilized for modeling.

**Fig 1:**
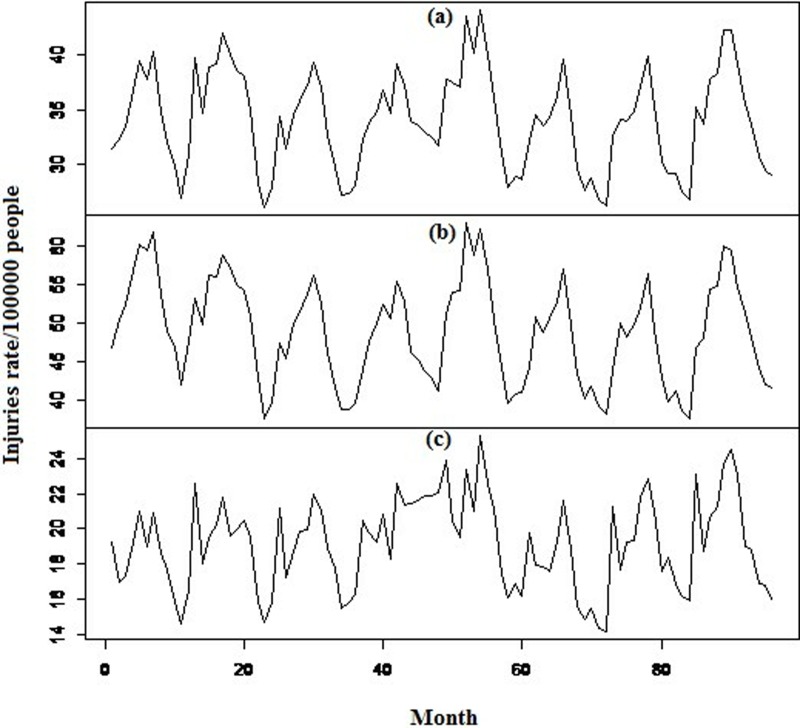
Monthly incidence rate of road traffic injuries in Iran: (a) total, (b) male, and (c) female, 2009–2016.

As shown in Fig 1, the RTIs were presented as an incidence rate. The IRTIs were calculated as the ratio of the number of injuries to the average monthly population (per 100,000 people per month). The monthly population was obtained through the aggregation of the previous month’s population with the number of births and deaths. Monthly births and deaths were available on the National Organization for Civil Registration website [28]. The average monthly population was also calculated from the average population at the beginning and the end of each month. Based on the reported data, annual values of IRTI increased from 398.37 (2015) to 426.86 (2016). However, as can be seen in Fig 1, in a longitudinal study of IRTI over the course of 12 years, trend reduction was not tangible.

### 2.2. Time series analysis

A time series is a sequence of observations. The intrinsic nature of a time series is the correlation of its observations. Using the auto-correlation and partial auto-correlation functions for a parametric study of a time series is termed time domain analysis. An alternative in this regard is frequency domain analysis, which performs a nonparametric decomposition study of a time series based on its various frequency components using spectral functions [29]. In this study, time domain analysis was employed for IRTI time series modeling. There are several methods for modeling in the time domain, including SARIMA and exponential smoothing methods, which are discussed below.

#### 2.2.1. SARIMA Model

The ARMA model contains autoregressive (AR) and moving average (MA) models. Combining differencing with an ARMA model yields a non-seasonal ARIMA model (p, d, q). One of the assumptions of the ARMA model is the stationarity of the time series. This assumption can include stationarity in the mean or variance. The stationarity property of the time series reflects independence of mean and variance from time. Thus, due to the effect of trend or season on its values at different times, the time series is not stationary. In general, a non-stationary time series is unpredictable in the long term [30]. Unit root tests (e.g., Dickey-Fuller’s test) are used to determine the stationarity [11].

Most time series in the field of epidemiology include a seasonal component. The final added complexity to the ARIMA model is seasonality. The ARIMA symbol can simply be generalized to consider the seasonal aspects and is presented as ARIMA (p, d, q) (P, D, Q)_s_, where s is the number of periods per season. A SARIMA model with s periods in each season is written as Eq 1 [11].

$$\left(1-\phi_{p}\right)\left(1-\Phi_{P}B^{S}\right)\left(1-B\right)\left(1-B^{S}\right)y_{t}=\left(1-\theta_{q}B\right)\left(1-\Theta_{Q}B^{S}\right)e_{t}$$ (1)

where p is the order of AR, d is the degree of first differencing, q is the order of MA, and P, D, and Q are related to the seasonal component of the model. In this formula, the backward shift operators of B^s^ and B represent differences with lag s and lag 1, respectively. The parameters D and d are equal to the number of seasonal and trend differences, respectively.

Box-Jenkins algorithm is used to determine the seasonal model [22]. The basic assumptions in using the Box-Jenkins method are stationarity in the variance and mean. At first, by observing the time series plot, when a decrease or increase occurs in the variance over time, various transformations (e.g., logarithmic transformation) should be performed on the original data. In this regard, to determine the linearity and stationarity, Keenan and Augmented Dickey-Fuller (ADF-GLS) tests can be used. In addition, ACF and PACF plots can be employed for detection of stationarity. If there is a lack of stationarity in the mean, seasonal or trend differences should be used. If a difference is required, a seasonal difference is performed initially, and again the ACF and PACF plots are checked for a required trend difference [11]. Upon detection of stationarity in the mean, by sample ACF and PACF, each of the parameters in the SARIMA model, including p, d, q, P, D, and Q, are estimated. AIC and BIC criteria are then used to compare candidate models for selecting the best option; that is, the model with the lowest AIC and BIC.

The main assumptions for accepting a SARIMA model are un-correlation, zero mean, and stationarity in the variance of the residuals. A time series model with such characteristics is termed white noise. Thus, the white noise model is a randomized model, where Y_t_ observations consist of two parts: a level term with a zero mean and a random error component that is uncorrelated from one time period to another. In addition to these two characteristics, other criteria such as normality and stationarity of variance in the residuals can be used as optional conditions. The ACF plots of residuals are applied in the final models to examine un-correlation [30].

Anderson [31], Bartlett [32], and Quenouille [33] showed that the sample auto-correlation coefficients of white noise data are estimated as a normal distribution with zero mean and a standard error of 1 / n^40^, where n is the number of observations in the series. At a significance level of 0.05, all auto-correlation coefficients must be within a range of mean ± 1.96*σ. Thus, in 20 lags in the ACF plots, a maximum of one sample autocorrelation coefficient should be more than the confidence interval. LB and Kolmogorov-Smirnov (KS) tests are also used for un-correlation and normality of residuals with a mean of zero and a variance of σ^2^. The significance level for all tests was considered to be 5%. The zero mean and stationarity in the variance of the residuals were observed in the plots. Finally, the prediction of IRTI time series outside the range of data utilized for modeling was validated by exponential smoothing methods, which will be discussed in the next section.

#### 2.2.2. Exponential smoothing methods

Exponential smoothing methods are employed in fitting classical models and prediction. In these methods, the components of trend, season, and error are smoothed with the help of proper relationships. Exponential smoothing methods always start with the trend component, which consists of a level term and a growth term. The level term and growth term can forecast the future trend in five modes according to Eqs 2–6 [34].

$$\text{None:}\;\;T_{h}\;=\;l$$ (2)

$$\text{Additive:}\;\;T_{h}\;=\;l+bh$$ (3)

$$\text{Additive}\;\text{damped:}\;\;T_{h}\;=\;l+(\phi +\phi^{2}+\ldots +\phi^{h})b$$ (4)

$$\text{Multiplicative:}\;\;T_{h}\;=\;lb^{h}$$ (5)

$$\text{Multiplicative}\;\text{damped}:T_{h}=lb^{\left(\phi +\phi^{2}+\ldots +\phi^{h}\right)}$$ (6)

where the terms *T*_*h*_, l, b, and φ are h periods forecasting in the trend, level, growth rate, and the damped trend parameter (0 <φ <1), respectively. In the damped trend method, the growth rate associated with the trend decreases over time [34].

In exponential smoothing methods, in addition to the trend component, there are also the seasonal and error components in an additive (A) or multiplicative (M) manner. If the error is ignored, there are 15 modes for combining the trend and season components in the exponential smoothing method, as shown in Table 1. This classification was initially proposed by Pegels [35] and was then extended by Gardner et al. [36]. Finally, Table 1 was modified by Hyndman et al. [37] and Taylor [38].

**Table 1:** Exponential smoothing methods.

Trend Component		Seasonal Component	
	N(None)	A(Additive)	M(Multiplicative)
N(None)	N,N	N,A	N,M
A(Additive)	A,N	A,A	A,M
Ad(Additive damped)	Ad,N	Ad,A	Ad,M
M(Multiplicative)	M,N	M,A	M,M
Md(Multiplicative damped)	Md,N	Md,A	Md,M

In this table, each of the trend and season components can be combined in a multiplicative or additive manner. Some of these methods have received more attention than others. For example, (N, N) represents a simple exponential smoothing, (A, N) represents the linear method of Holt, and (Ad, N) represents the damped trend method. The additive and multiplicative Holt-Winters methods are, respectively, (A, A) and (A, M). In the following, the additive Holt-Winters method (A, A) is represented by Eqs 7–10, because of its application in this study [34].

$$\ell_{t}=\;\alpha \left(y_{t}-s_{t-m}\right)+\left(1-\alpha \right)\left(\ell_{t-1}+b_{t-1}\right)$$ (7)

$$b_{t}=\beta^{*}\left(\ell_{t}-\ell_{t-1}\right)+\left(1-\beta^{*}\right)b_{t-1}$$ (8)

$$s_{t}=\gamma \left(y_{t}-\ell_{t-1}-b_{t-1}\right)+\left(1-\gamma \right)s_{t-m}$$ (9)

$${\hat{y}}_{t+h|t}=\ell_{t}+b_{t}h+s_{t-m+h_{m}^{+}}$$ (10)

where m, $\ell_{t}$, b_t_, s_t_, and ${\hat{y}}_{t+h|t}$ are the length of season, level of time series, growth rate, season, and forecasting h periods in the future. $h_{m}^{+}$ in formula 15 is obtained from $\;h_{m}^{+}\;=\;\left[\left(h-1\right)mod\;m\right]+1$. *α*, *β*, and *γ* are the weights of the level, the growth rate of the trend, and the season of time series, respectively. These weights always have a constant value between zero and one, indicating participation in the predicted values. For example, if β is non-significant, the trend parameter is considered to be zero. These weights are obtained by minimizing the AIC value or other criteria.

For each of the modes in Table 1, the error can also be combined in an additive or multiplicative manner, known as state-space methods. This method is always displayed as (Z, Z, Z), each consisting of error, trend, and season, respectively. Although trend and season consist of additive, multiplicative, and none, the error only contains additive or multiplicative components. Therefore, taking into account the error component in the state-space methods, 30 models were obtained for exponential smoothing methods. It is necessary to distinguish between the state-space and exponential smoothing methods [34]. In the present study, R 3.4.3 software was employed for modeling time series [39].

## 3. Results and discussion

### 3.1. SARIMA model

**Fig 2:**
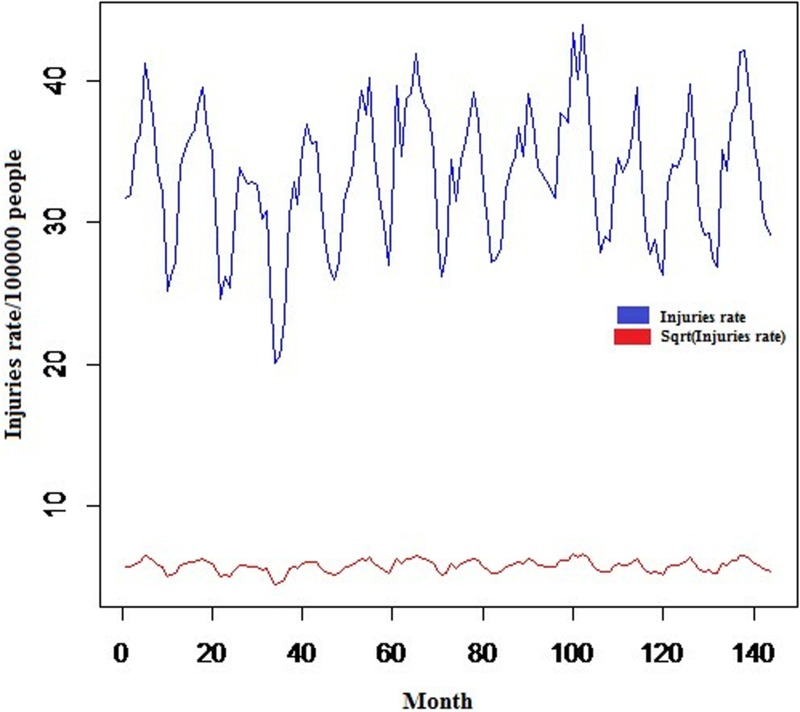
Original (blue line) and transformed (red line) temporal distribution of monthly incidence rate of total road traffic injuries in Iran, 2005–2016.

In this section, first, the stationarity analysis of variance is investigated. The approach is to achieve stationarity in the variance through a transformation over a time series. Regarding Fig 1, the IRTI and the IRTI of females were considered non-stationary during the study period. Therefore, the square root and logarithmic transformations were used for the IRTI and the IRTI of females, respectively. For example, stationarity in the variance of IRTI is evident in Fig 2, where the original time series is observed along with its logarithmic transformation. The IRTI of males and males-to-females remained unchanged, due to the observation of stationarity in the variance.

After the variance, mean stationarity was studied. According to Fig 1, non-stationarity in the mean was observed in almost all IRTI time series. In addition, Keenan and ADF-GLS tests were used to determine linearity and stationarity. The p-value of these tests was calculated in Table 2 before and after logarithmic and square root transformation and with differences of 12 and 1 lags. For instance, the hypotheses of mean stationarity and linearity of IRTI of females were rejected according to the p-values; however, these hypotheses were accepted after the difference at lag 1. The ACF and PACF plots related to the converted time series were used for mean stationarity detection.

**Table 2:** Results of statistical tests for the linearity and stationarity.

Model		Keenan test	ADF-GLS test
**IRTI**	Total Injuries	0.425	<0.01
	Diff(1,12) (sqrt^*^(Injuries))	0.041	<0.01
**IRTI of males**	Male Injuries	0.017	<0.01
	Diff (1,12)(Male Injuries)	0.635	0.02
**IRTI of females**	Female Injuries	0.002	0.39
	Diff (1)(log^**^(Female Injuries))	0.102	<0.01
**IRTI of males-to-females**	Male/Female Injuries	0.031	0.095

*square root,

**logarithmic

The reason for the differences in lag 1 and 12 for IRTI and IRTI of males, despite the acceptance of the mean stationarity hypothesis before differencing, was the un-correlation in the residuals of the final models as one of the main assumptions in the SARIMA method.

In order to achieve mean stationarity, at first if the seasonal component was observed in the ACF plot, the seasonal difference was made with a lag 12. If the stationarity was then not achieved, the difference in the trend was made at lag 1. Due to the monthly withdrawal of data, parameter *s*, which is the number of periods per season, was 12.

**Fig 3:**
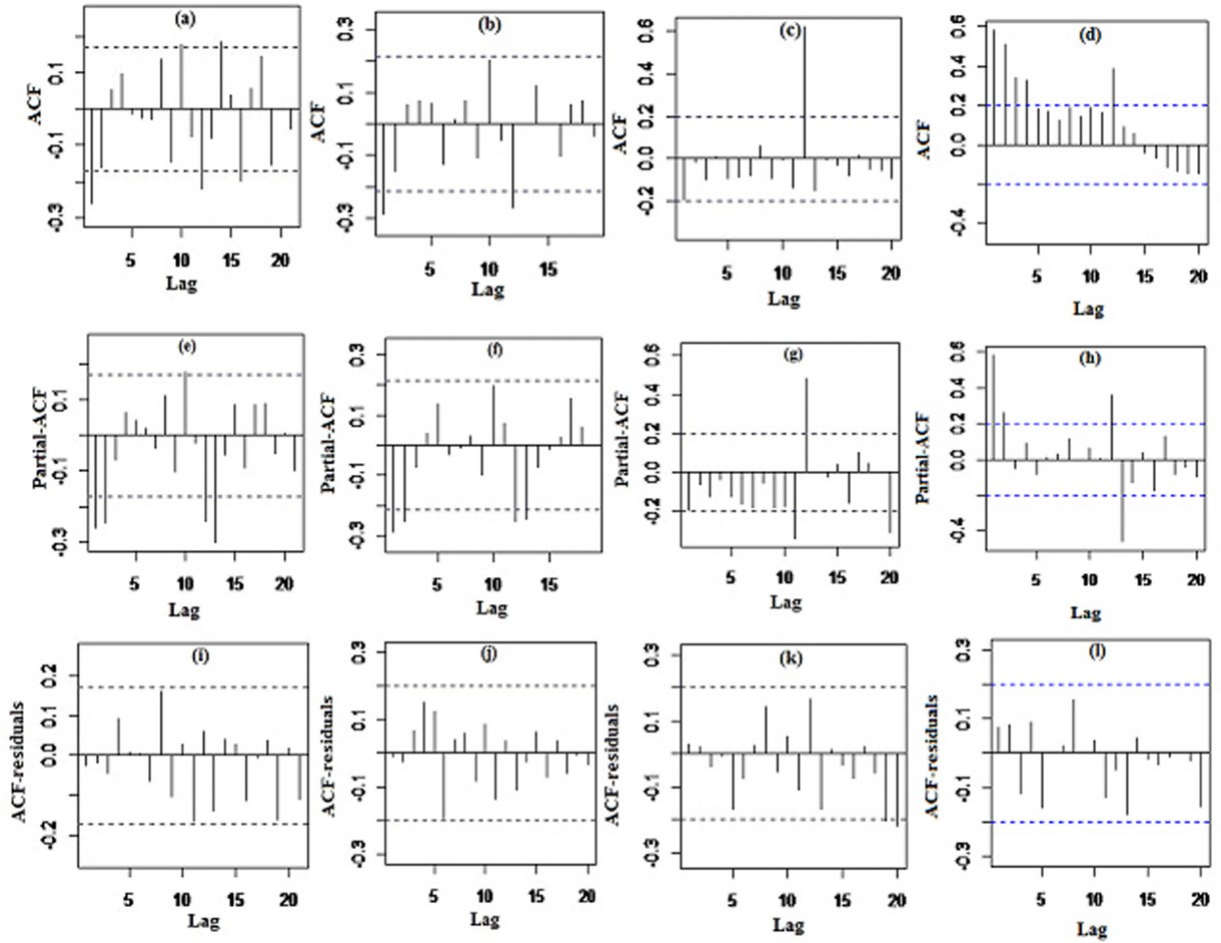
ACF, partial ACF after differences, and ACF of residuals of monthly incidence rate of (a,e,i)total road traffic injuries (from 2005 to 2016), and monthly incidence rate of (b,f,j)males, (c,g,k)females and (d,h,l)male/female road traffic injuries(from 2009 to 2016) in Iran.

In order to determine the order of (p, d, q) (P, D, Q)_s_ in the final SARIMA models, ACF and PACF plots, shown in Fig 3, were used. Table 2 was considered to obtain parameters d and D (differences at lag 1 and lag 12). Sample partial autocorrelation values for partial-ACF(e-h) plots were applied to estimate p and P. In order to obtain p, PACF must be significant until lag p in the partial-ACF plot. In addition, to obtain P, PACF values must be significant at lag 12. Estimation of q and Q was similar to p and P, except that ACF plots(a-d) were used in the determination.

Table 3 presents AIC and BIC values for the SARIMA models. Considering the significance of the values of ACF and PACF in Fig 3, different SARIMA models were selected as the candidates for the best model. The lowest AIC and BIC were the criteria taken into account when selecting the best model. For instance, the SARIMA (2,1,0) (0,1,1)_12_ was chosen for the IRTI with an AIC and BIC of 84.57 and -71.44, respectively.

**Table 3:** AIC and BIC values for the different SARIMA models.

Model	AIC	BIC
**(a)Total Injuries**		
(2,1,0) (1,1,0)	-70.68	-59.71
(2,1,0) (0,1,1)	-84.57	-71.44
**(b) Male**		
(2,1,0)(1,1,1)	415.92	428.02
(2,1,0) (0,1,1)	415.12	424.8
(0,1,1)(1,1,1)	414.51	424.19
(0,1,1)(1,1,0)	420.6	427.86
(0,1,1)(0,1,1)	413.38	420.63
**(c) Female**		
(1,1,1)(0,0,1)	-177.35	-165.13
(1,1,1)(0,0,1)	-166.8	-157.14
(0,1,1)(0,0,1)	-167.17	-157.51
**(d) Male/Female**		
(2,0,0)(1,0,0)	-85.32	-70.5
(1,0,0)(1,0,0)	-83.49	-68.67

SARIMA(0,1,1)(0,1,1)_12_, SARIMA (1,1,1) (0,0,1)_12_, and SARIMA(2,0,0)(1,0,0)_12_ models were selected for IRTI of males, females, and males-to-females, respectively. In some models, such as IRTI, although the lag 1 in the ACF was statistically significant, lack of parameter significance at a significance level of 0.05 resulted in non-selection of parameter q in each of the candidate models.

The parameters estimated for the final SARIMA models based on the minimum AIC values are shown in Table 4. This table contains the parameters of the SARIMA models, standard errors, z- and p-values. With regard to p-value at a 0.05 significance level, all parameters in each of the final models were significant. Lack of correlation, zero mean, and stationarity in the variance of residuals were investigated for white noise. According to the p-value of the LB test in Table 5, the hypothesis of the un-correlation of the residuals for all models was accepted. In addition to the LB test, ACF(i-l) plots for the residuals were used in Fig 3. Near zero autocorrelation values indicated un-correlation, which was clearly evident in each of these plots. In addition, zero mean and stationarity in the variance were observed in the residuals plot. In the SARIMA method, in addition to the white noise, the hypothesis of normality was also accepted by the results of the KS test in Table 5. Table 5 presents the results of statistical tests for exponential smoothing, including the state-space and Holt-Winters methods, which are discussed in the following section.

**Table 4:** Results of parameter estimation for the SARIMA models.

Model	Parameter	Standard error	z	p-value
**(a) Total Injuries**				
Non-seasonal lags				
AR1	-0.435	0.088	-4.931	8.191E-07
AR2	-0.265	0.089	-2.975	0.003
Seasonal lags				
MA1	-0.597	0.095	-6.265	3.73E-10
**(b) Male**				
Non-seasonal lags				
MA1	-0.503	0.089	-5.669	1.433E-08
Seasonal lags				
MA1	-0.679	0.139	-4.88	1.062E-06
**(c) Female**				
Non-seasonal lags				
AR1	0.684	0.078	8.829	< 2.2e-16
MA1	-1	0.0267	-37.528	< 2.2e-16
Seasonal lags				
MA1	0.631	0.088	7.183	6.845e-13
**(d) Male/Female**				
Non-seasonal lags				
AR1	0.538	0.1	5.395	6.85e-08
AR2	0.253	0.099	2.548	0.011
Seasonal lags				
AR1	0.694	0.073	9.479	< 2.2e-16
Intercept	2.641	0.176	14.993	< 2.2e-16

**Table 5:** Results of statistical tests for residuals of final models.

Model	Injuries	Male Injuries	Female Injuries	Male/Female
	p-value			
**SARIMA**				
LB	0.539	0.3	0.164	0.237
KS	0.372	0.722	0.083	0.247
**State-Space**				
LB	0.323	0.256	0.02	0.005
KS	0.91	0.949	0.021	0.206
**Holt-Winters**				
LB	0.606	0.531	0.005	0.004
KS	0.902	0.847	0.777	0.376

### 3.2. Exponential smoothing methods

To validate the prediction of SARIMA models outside the studied range, exponential smoothing methods including Holt-Winters and state-space were employed. In this study, the best models in each of these methods were selected on the basis of the lowest AIC.

Regarding modeling by exponential smoothing methods, β was statistically non-significant for all the time series except for IRTI of males-to-females. The non-significance of β suggests lack of any trend (Fig 1). The Holt-Winters model (N, A) was used with level and seasonal components for all the time series. In the state-space method, (A, N, A), (A, N, A), (M, N, A), and (A, N, A) models were chosen for IRTI, IRTI of males, females and males-to-females, respectively. Table 6 contains the estimation of α, β, and γ parameters for the state-space and Holt-Winters models.

**Table 6:** Parameters estimated for the state-space and Holt-Winters models.

Model	Type	alpha	beta	gamma
**State-Space**				
Injuries	A,N,A	0.471	0	0.0001
Male	A,N,A	0.48	0	0.0001
Female	M,N,A	0.5229	0	0.0001
Male/Female	A,N,A	0.492	0	0.0001
**Holt-Winters**				
Injuries	(N,A)	0.444	0	0.527
Male	(N,A)	0.407	0	0.736
Female	(N,A)	0.579	0	1
Male/Female	(N,A)	0.624	0.004	1

According to the LB results in Table 5 for the Holt-Winters and state-space methods, the hypothesis of un-correlation was acceptable only for the IRTI and IRTI of males. Note that the assumption of residuals being white noise in the Holt-Winters model, which included trend and season parameters, was not considered as the principal acceptance condition of the model. However, this was the case in the state-space method, due to the presence of the error component in the model [34]. Based on the KS test, the hypothesis of normality as an optional condition was accepted for all time series in the state-space method, except for IRTI of females.

### 3.3 Prediction

**Fig 4:**
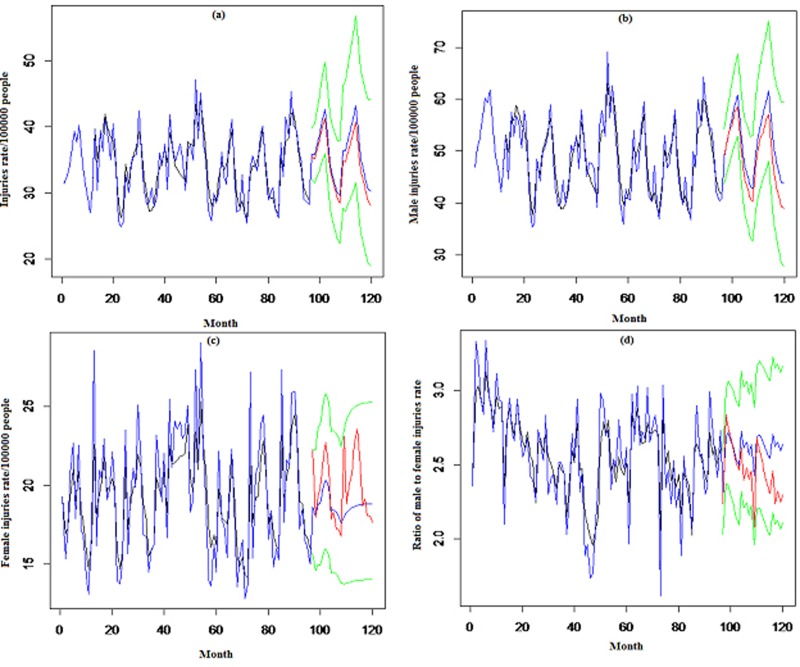
Predicted(SARIMA: blue line, Holt-Winters: red line), actual (black line), and 95CI (green line) monthly incidence rate of (a) total, (b) male, (c) female, and (d) male/female road traffic injuries in Iran, 2009–2016.

The actual (black line) and fitted (blue line) values obtained from SARIMA models for all the time series are shown in Fig 4. The plots in Fig 4A–4D correspond to IRTI, IRTI of males, females, and males-to-females, respectively. The blue line, in addition to the fitted values, presents the predictions of the SARIMA model for the next 24 months (from 2016 to 2018) based on the behavior of trend and season components of the main data. The blue lines in all the time series resulted in almost no decrease or increase in trend. The green lines are the 95% confidence intervals (95CI) of the prediction. The red lines in Fig 4, which represent the predicted values by using the Holt-Winters method, were utilized to validate the behavior of the SARIMA models in the prediction for the next 24 months. The red lines confirmed the behavior of SARIMA models from 2016 to 2018.

## 4. Conclusions

This study recommends the use of a SARIMA model to predict IRTI, IRTI of males, females, and males-to-females in Iran. The prediction of the SARIMA model for the IRTI time series can influence traffic safety policymaking in the future by simulating the impact of future interventions. One of the advantages of the SARIMA model is the non-use of complex transformations or external variables due to operations, such as difference, autoregressive, moving average, and seasonal functions for trend, auto-correlation, smoothing, and season [10].

The assumptions of the SARIMA model include stationarity in the variance and mean of the actual data. Studies conducted by Box and Jenkins suggest the use of seasonal and trend differences for non-stationarity in mean and transformations, including the logarithm for non-stationarity in the variance [10]. In this study, ADF-GLS and Keenan tests were applied to examine the hypothesis of stationarity in the mean and linearity at a 0.05 level of significance. In addition, time series plots and ACF / PACF plots were utilized to detect the stationarity of variance and mean, respectively.

White noise was one of the main assumptions for acceptance of SARIMA models. Un-correlation and zero mean were the prerequisites for accepting white noise in the residuals. The ACF and PACF plots showed the un-correlation. The results of the LB test confirmed the un-correlation in the modeling. In addition, zero mean and the stationarity of variance of the residuals were also observed in the plots. The normality and stationarity of the variance were evaluated and satisfied using a KS test and the residuals plot, respectively.

SARIMA (2.1.0) (0.1.1)_12_, SARIMA (0.1.1) (0.1.1)_12_, SARIMA (1.1.1) (0.0,1)_12_, and SARIMA (2,0,0) (1,0,0)_12_ were obtained for the IRTI, IRTI of males, females, and males-to-females in Iran, respectively. The validity of SARIMA models for IRTI time series was confirmed in out-of-range predictions by exponential smoothing methods, such as Holt-Winters and state-space. Adequate conformity was observed in training the SARIMA models on actual data in the studied range.

The decreasing trend of IRTI of males was observed for 2009–2010, but then no change was observed for the study period and the predicted values until 2018. An increase in the trend of IRTI of females occurred for 2009–2012; showing a likely growth in the number of women who obtained a driving license, reflecting an increase in social status of women. This increase, however, was neutralized by a decline from mid-2013. In addition, the trend in prediction values did not change.

The observed and predicted trends of IRTI of males-to-females during the study period illustrated that males were injured two to three times more than females, due to the fact that men were more active outside the home, and utilized more modes of transportation than women. This fact resulted in more men in traffic crashes in Iran. The prediction trend of IRTI of males-to-females did not change, due to no reduction in the trend.

The predicted values of IRTI by the SARIMA model showed no decreasing trend until 2018. Thus, applying interventions as traffic legislation when using interrupted time series simulation would help inform policymaking to reduce IRTIs in the future.

## Supporting information

S1 File Total aggregated data-injuries. (ZIP)

## Abbreviations

IRTI: Incidence rates of total Road Traffic Injuries
RTI: Road Traffic Injuries
RTM: Road Traffic Mortality
SARIMA: Seasonal Auto-Regressive Integrated Moving Average
AIC: Akaike Information Criterion
BIC: Bayesian Information Criterion
LB: Ljung-Box
ACF: Auto-Correlation Function
PACF: Partial Autocorrelation Function
